# Complete Mitochondrial Genome of *Suwallia teleckojensis* (Plecoptera: Chloroperlidae) and Implications for the Higher Phylogeny of Stoneflies

**DOI:** 10.3390/ijms19030680

**Published:** 2018-02-28

**Authors:** Ying Wang, Jin-Jun Cao, Wei-Hai Li

**Affiliations:** Department of Plant Protection, Henan Institute of Science and Technology, Xinxiang 453003, Henan, China; wangying198586@163.com (Y.W.); cjj1986108@163.com (J.-J.C.)

**Keywords:** Plecoptera, Chloroperlidae, *Suwallia teleckojensis*, mitochondrial genome, phylogenetics

## Abstract

Stoneflies comprise an ancient group of insects, but the phylogenetic position of Plecoptera and phylogenetic relations within Plecoptera have long been controversial, and more molecular data is required to reconstruct precise phylogeny. Herein, we present the complete mitogenome of a stonefly, *Suwallia teleckojensis*, which is 16146 bp in length and consists of 13 protein-coding genes (PCGs), 2 ribosomal RNAs (rRNAs), 22 transfer RNAs (tRNAs) and a control region (CR). Most PCGs initiate with the standard start codon ATN. However, *ND5* and *ND1* started with GTG and TTG. Typical termination codons TAA and TAG were found in eleven PCGs, and the remaining two PCGs (*COII* and *ND5*) have incomplete termination codons. All transfer RNA genes (tRNAs) have the classic cloverleaf secondary structures, with the exception of *tRNA^Ser(AGN)^*, which lacks the dihydrouridine (DHU) arm. Secondary structures of the two ribosomal RNAs were shown referring to previous models. A large tandem repeat region, two potential stem-loop (SL) structures, Poly N structure (2 poly-A, 1 poly-T and 1 poly-C), and four conserved sequence blocks (CSBs) were detected in the control region. Finally, both maximum likelihood (ML) and Bayesian inference (BI) analyses suggested that the Capniidae was monophyletic, and the other five stonefly families form a monophyletic group. In this study, *S. teleckojensis* was closely related to *Sweltsa longistyla*, and Chloroperlidae and Perlidae were herein supported to be a sister group.

## 1. Introduction

In metazoans, the mitochondrial genome (mitogenome) is usually a circular, double-stranded molecule, ranging in size from 13 to 16 kb [1,2]. It contains a remarkably conserved set of 37 genes, i.e., 13 protein coding genes (PCGs), 2 ribosomal RNA (rRNA), and 22 transfer (tRNA) genes [1,2]. Additionally, an A + T-rich region is known as the non-coding region or the control region (CR), which is involved in the initiation of transcription and replication [2]. Because of their abundance, small size, fast rate of evolution, and low levels of sequence recombination, mitochondrial genomes are increasingly applied to comparative and molecular evolution, phylogenetic studies, and population genetics [3].

The Plecoptera (stoneflies) is a small order of hemimetabolous insects. It is comprised of 16 families and includes about 3900 described species worldwide [4,5]. Chloroperlidae includes more than 200 species mainly in the Holarctic region [5]. The chloroperlid genus *Suwallia* Ricker, 1943 is primarily distributed in North America, Japan, Russia, and Mongolia [6,7]. Recently, four species of the chloroperlid genus *Suwallia*, *S. teleckojensis*, *S. decolorata*, *S. talalajensis*, and *S. wolongshana* are reported from China [8,9,10].

Stoneflies comprise an ancient group of insects, but the phylogenetic position of Plecoptera has long been controversial [11]. There are different opinions based on morphological data [12,13,14], and later, molecular data makes these opinions more controversial [15,16,17]. In addition, phylogenetic relations within Plecoptera, such as, Styloperlidae, Peltoperlidae, Chloroperlidae, Perlidae, and Perlodidae was controversial [18,19,20,21]. Currently, the position of Pteronarcyidae, Styloperlidae, and Peltoperlidae in Pteronarcyoidea has been resolved based on morphology [19], and our previous study that analyzed the family-level phylogenetic of the Pteronarcyoidea supported the traditional morphology-based classification [22]. However, other recent analyses based on the mitogenomic data still do not support the traditional classification well [23,24,25]. Coincidentally, the position of Chloroperlidae, Perlidae, and Perlodidae was proposed based on morphological data [26,27], but more conflicting opinions were raised after molecular data became available. For example, most studies supported Chloroperlidae and Pteronarcyidae as a sister-group [20,21,24,25,28,29,30]. Up to now, only one chloroperlid species, *Sweltsa longistyla* was sequenced [31], and those conflicting opinions were mainly generated by the limited mitogenomic data. Therefore, more molecular data is required to reconstruct precise phylogeny [27,32,33].

To date, eighteen complete and four nearly complete mitogenomes of stoneflies have been sequenced [20,21,22,24,25,28,29,30,31,32,34,35,36,37,38,39,40,41]. In this article, we provided the second mitogenome of the stonefly family Chloroperlidae to facilitate a study of mitochondrial phylogeny in the Plecoptera. We sequenced the mitogenome of *S. teleckojensis* and analyzed the nucleotide composition, codon usage, compositional biases, secondary RNA structures, stem-loop (SL) structures, and conserved sequence blocks (CSBs) in the control region. Furthermore, we also reconstructed the phylogenetic tree of *S. teleckojensis* and other stoneflies based on PCGs, thus our result increases the understanding of stonefly phylogeny.

## 2. Results and Discussion

### 2.1. Genome Organization and Base Composition

**Figure 1:**
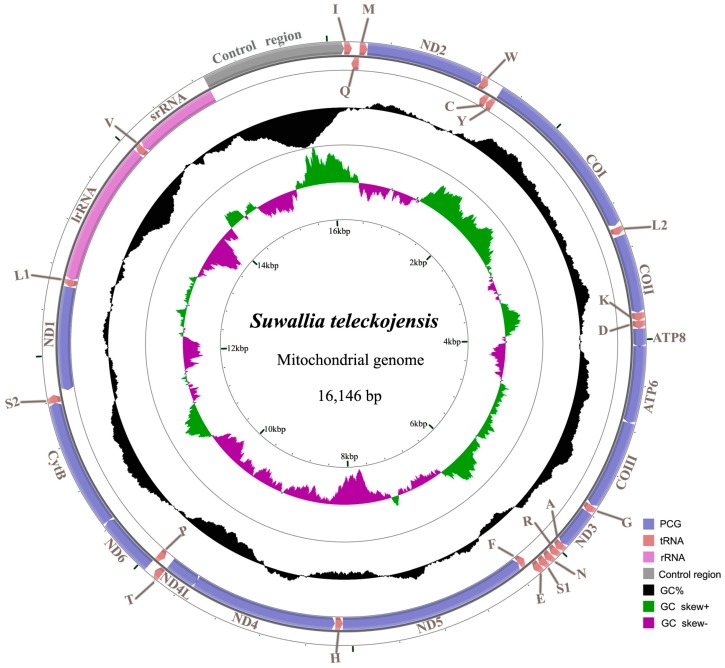
Map of the mitochondrial genome of *S. teleckojensis*. Direction of gene transcription is indicated by the arrows. Protein-coding genes (PCGs) are shown as blue arrows, rRNA genes as purple arrows, tRNA genes as red arrows, and control region (CR) as gray arrows. tRNA genes are labeled according to single-letter IUPAC-IUB abbreviations (L1: UUR, L2: CUN, S1: AGN, S2: UCN). The GC content is plotted using a black sliding window, as the deviation from the average GC content of the entire sequence. GC skew is plotted as the deviation from the average GC skew of the entire sequence.

The complete mitogenome of *S. teleckojensis* was 16,146 bp in length (Figure 1), which was nearly identical to the genome of *S. longistyla* (16,151 bp) and similar in length with the other previously sequenced completely mitogenomes of plecopteran insects (Table 1). It contains 37 genes (including 13 PCGs, 22 tRNAs, and 2 rRNAs) and a control region. Most genes (23 genes) were located on the J-strand (major strand), the remaining being oriented on the N-strand (minor strand) [1]. In addition to the control region, there were 76 nucleotides dispersed in 14 intergenic spacers, ranging from 1 to 20 bp. The longest non-coding intergenic spacer (20 nucleotides) was located between *ND1* and *tRNA^Leu(CUN)^*. Gene overlaps were also found at 11 gene junctions involving 41 nucleotides with the longest overlaps (8 nucleotides) between *tRNA^Cys^* and *tRNA^Tyr^*, and *COI* and *tRNA^Leu(UUR)^* (Figure 1 and Table 2).

The gene order of *S. teleckojensis* is identical to *Drosophila melanogaster* Meigen, which is considered to be the typical arrangement of the inset mitochondrial genes [1]. All genes have similar locations and strands to those of other published stoneflies. The arrangement of mitochondrial genes of *S. teleckojensis* is the same as *S. longistyla* [31] and should be conservative in all sequenced stoneflies.

The nucleotide composition of the mitogenome was biased toward A and T, with 66.7% of A + T content (A = 36.5%, T = 30.2%, C = 21.7%, G = 11.6%). The A + T content of PCGs, tRNAs, rRNAs, and control region is 64.1%, 69.2%, 71.5%, and 79.1%, respectively (Table 3). To evaluate the degree of the base bias, we measured base-skew. The whole genome displayed negative GC-skew and positive AT-skew. The PCGs, J-strand of PCGs, N-strand of PCGs, N-strand of tRNAs and rRNAs had a negative AT-skew, while the N-strand of tRNAs had a positive AT-skew. The tRNAs in the J-strand displayed positive AT-skews, whereas the N-strand showed negative AT-skews. This feature was probably related to the asymmetrical directional mutation pressure [42].

### 2.2. Protein-Coding Genes and Codon Usage

The total length of all 13 PCGs of *S. teleckojensis* was 11,244 bp. All but two of the PCGs in *S. teleckojensis* utilize the conventional translational start codons for invertebrate mitochondrial DNA (mtDNA). For example, one PCG (*COI*) contained an ATT codon, two PCGs (*ND3* and *ND6*) contained ATC codons, and eight PCGs (*ND2*, *COII*, *ATP8*, *ATP6*, *COIII*, *ND4*, *ND4L,* and *CytB*) initiated with ATG codons. Two exceptions were *ND5* and *ND1*, which used GTG and TTG as a start codon, respectively, as reported for most other Plecoptera [22,30]. Eleven PCGs used the typical termination codons TAA and TAG in *S. teleckojensis*, while only two PCGs (*COII* and *ND5*) stopped with the incomplete termination signal T. The stop codons TAA and TAG always had an overlap comprising several nucleotides with the downstream tRNAs (Table 2), which was supposed to act as a “backup” to prevent translation read-through if the transcripts were not properly cleaved [43]. The presence of incomplete stop codons is a feature shared with many arthropod mitochondrial genomes [2] and these truncated stop codons were assumed to be completed post-transcriptionally by the polyadenylation of mature mRNA [44].

In many insect mitogenomes, the *ATP8*/*ATP6* and the *ND4L*/*ND4* gene pairs overlap by seven nucleotides (ATGNTAA) which are thought to be translated as a bicstron [45]. In the *S. teleckojensis* mitogenome, the overlap nucleotides were conserved for *ATP8*/*ATP6* (ATGATAA) and *ND4L*/*ND4* (ATGTTAA).

The relative synonymous codon usage (RSCU) reflects the influence of strongly biased codon usage [46]. The wide base compositional biases of the genome for AT was well reflected in the codon usage. There is a strong bias toward AT-rich codons with the four most prevalent codons in *S. teleckojensis* in the order: TTA (Leu), ATT (Ile), TTT (Phe), and ATA (Met) (Table 4). The four most prevalent codons in *S. teleckojensis* were similar to those in *Capnia zijinshana* [28]. Molecular processes, such as translational selection efficiency and accuracy, may influence the codon usage. They apparently have a stronger influence in organisms with rapid growth rates [47].

### 2.3. Transfer RNAs

**Figure 2:**
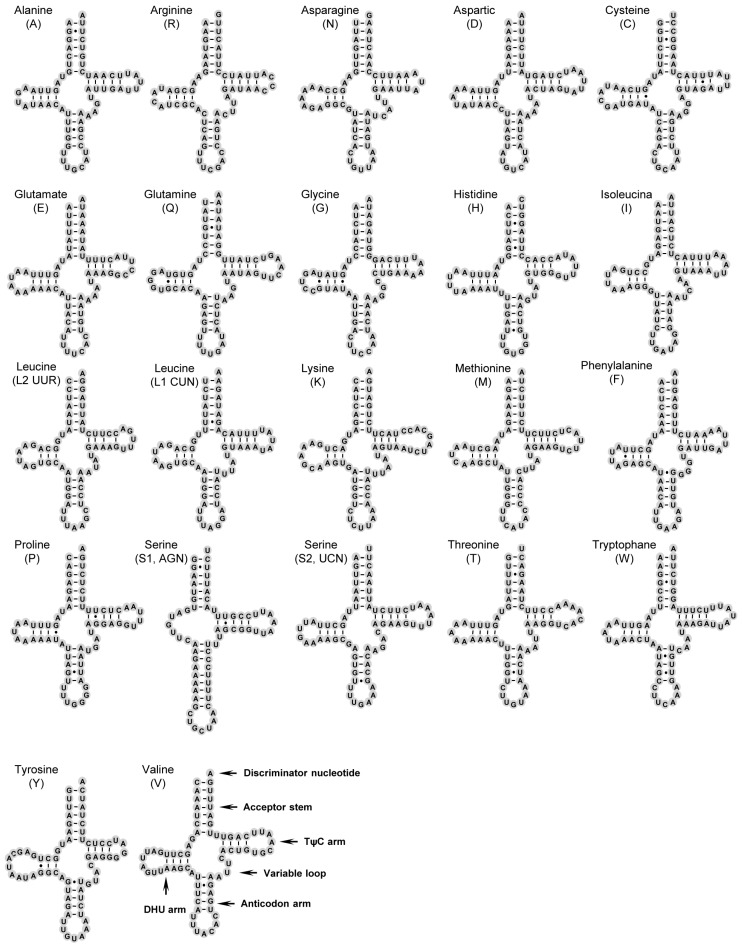
Secondary structures of 22 tRNAs of *S. teleckojensis*. All tRNAs are labeled with the abbreviations of their corresponding amino acids. Dashes (–) indicate Watson−Crick base pairing and dots (•) indicate G−U base pairing.

Twenty-two typical tRNAs in the arthropod mitogenomes were found in *S. teleckojensis* and their respective secondary structures are shown in Figure 2. All of tRNA genes could be folded as typical cloverleaf structures except for *tRNA^Ser(AGN)^*, in which its dihydrourine (DHU) could only form a loop (8 bp) with the DHU stem loss. In general, this phenomenon is a common condition in metazoan mtDNAs [48].

The length of *S. teleckojensis* tRNAs ranged from 65 to 71 bp (Table 2), similar to that of *S. longistyla* [31]. Like most of reported insect tRNAs, the lengths of the aminoacyl (AA) stem (7 bp), the anticodon (AC) stem (5 bp), and the AC loop (7 bp) of *S. teleckojensis* were invariable (Figure 2). The lengths of DHU arms and TΨC arms were more variable, ranging from 3 to 5 bp. The length of the AC stems was always conservative except that *tRNA^Ser(AGN)^* had the longest base pairing of all 22 tRNAs.

A total of 38 unmatched base pairs were identified in the stems of 17 different tRNAs. Compared with other stoneflies, most of the mismatched nucleotides were G–U pairs (29 base pairs), which can form a weak bond in tRNAs and non-canonical pairs in tRNA secondary structures [49]. These contained G–U pairs in amino acid arms (9 bp), DHU arms (7 bp), anticodon arms (10 bp), and TΨC arms (3 bp). The other unmatched base pairs were two U–U pairs in the AC stem of *tRNA^Ala^* and *tRNA^Tyr^*, three A–C pairs in the TΨC stem of *tRNA^Leu(UUR)^* and in the AA stem of *tRNA^Arg^* and *tRNA^Ser(AGN)^*, three A–A pairs in the AA stem of *tRNA^Gly^*, *tRNA^Leu(UUR)^* and *tRNA^Ser(UCN)^*, and one U–C pair in the AA stem of *tRNA^Met^* (Figure 2).

### 2.4. Ribosomal RNAs

Like other insect mitogenomes, the large and small rRNA subunits (*lrRNA* and *srRNA*) in *S. teleckojensis* were located at *tRNA^Leu(CUN)^*, *tRNA^Val^*, and *tRNA^Val^*—the control region, respectively (Figure 1 and Table 2). The lengths of *lrRNA* and *srRNA* were 1329 and 800 bp, respectively.

**Figure 3:**
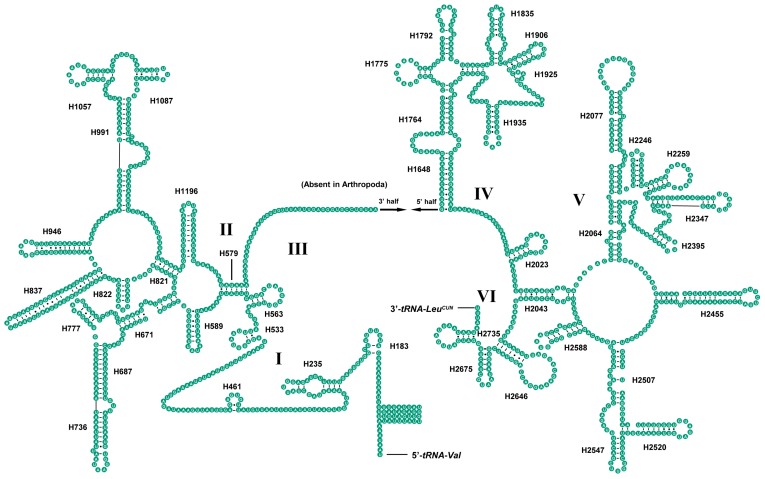
Predicted secondary structure of the *lrRNA* gene in *S. teleckojensis*. Roman numerals represent the conserved domain structures. Dashes (–) indicate Watson–Crick base pairings and dots (•) indicates G–U base pairing. I–VI indicate six domains in the secondary structure of *lrRNA*.

The secondary structures of both *lrRNA* and *srRNA* were drawn based on other insect models [22,50]. The secondary structure of *lrRNA* of *S. teleckojensis* largely resembled previously published structures for *Styloperla spinicercia* (Linnaeus, 1763) (Insecta: Plecoptera: Styloperlidae) [22]. It consisted of 5 structural domains (I, II, IV–VI) with domain III absent, which is a typical trait in arthropods (Figure 3) [51]. Compared with *S. spinicercia*, domains I, II, and IV were variable, whereas 5 helices (H2455, H2507, H2520, H2547, and H2588) within domain V had the highest similarity [22].

**Figure 4:**
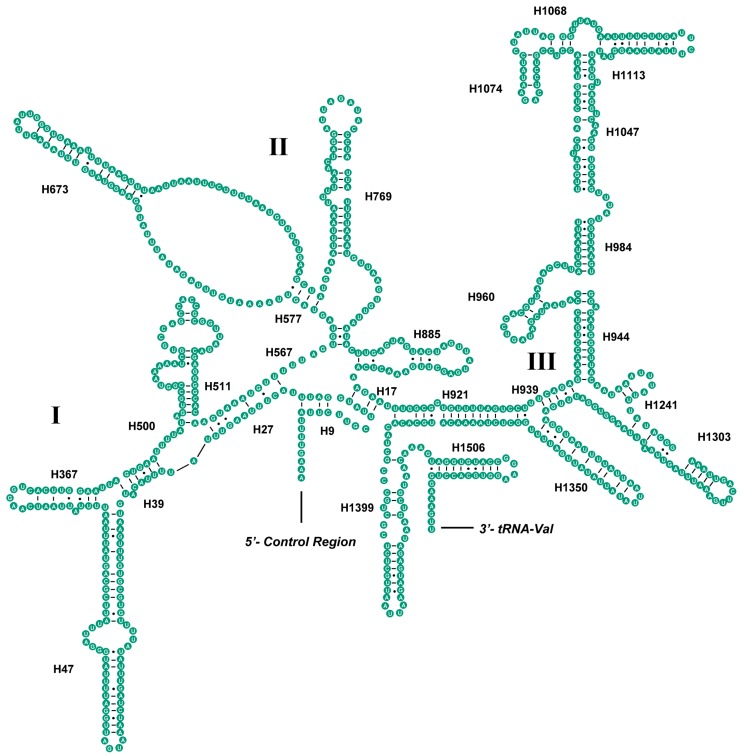
Predicted secondary structure of the *srRNA* gene in *S. teleckojensis*. Roman numerals denote the conserved domain structure. Dashes (−) indicate Watson-Crick base pairing and dots (•) indicate G–U base pairing. I–III indicate three domains in the secondary structure of *srRNA*.

The secondary structure of *srRNA* contained three domains (Figure 4). Similar to *S. spinicercia*, domain I was more variable than domains II and III, whereas Helix 1399 was the most conserved region [22]. In addition, *S. teleckojensis* possessed more nucleotides in Helix1068, Helix1074, and Helix 577.

We also compared the *lrRNA* and *srRNA* of *S. teleckojensis* with *S. longistyla*. The secondary structure of *lrRNA* and *srRNA* had high similarity (pair-wise sequence identity was 86.77% and 90.10%, respectively) and they should be conservative in the family Chloroperlidae.

### 2.5. The Control Region

**Figure 5:**
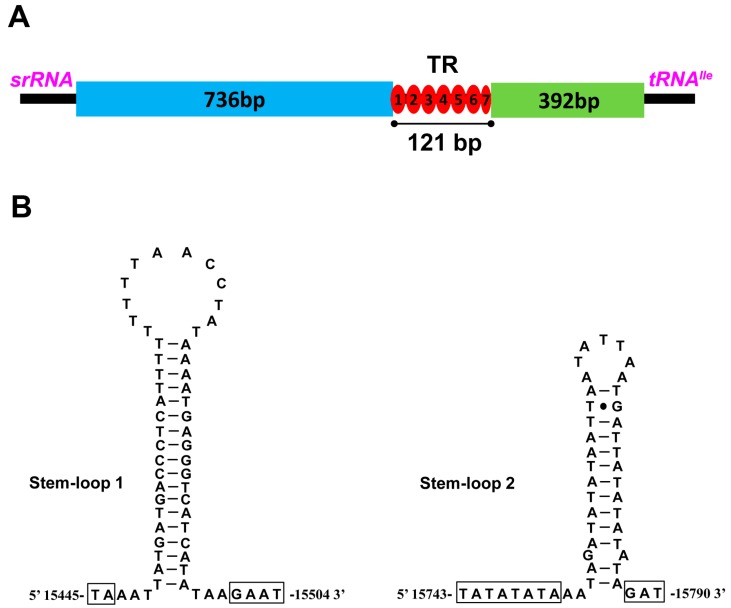
Control region of the *S. teleckojensis* mitogenome. (**A**) Structure elements found in the control region of *S. teleckojensis*. (**B**) Putative stem-loop structures found in the control region of *S. teleckojensis*.

Previously, the A + T-rich region, also called the control region (CR) was reported to contain elements essential to the initiation of replication and transcription [52]. The A + T-rich region of the *S. teleckojensis* mitogenome was 1249 bp in size, possessed an A + T content of 79.1%, and mapped between the *srRNA* and *tRNA^Met^* gene cluster (Figure 1). The control region in the *S. teleckojensis* mitogenome was longer than most other stoneflies and the A + T content was slightly lower than that of *S. longistyla* (80.1%). The following structural elements were summarized in the control region of *S. teleckojensis* mitogenome: (1) a leading sequence (736 bp, containing SL1 and poly-T) adjacent to *srRNA*, (2) a tandemly repeated sequence block consisting of six complete and one incomplete tandem repeat units (7 bp), and (3) the remainder of the control region (392 bp, containing SL2 and poly-N) (Figure 5A).

**Figure 6:**
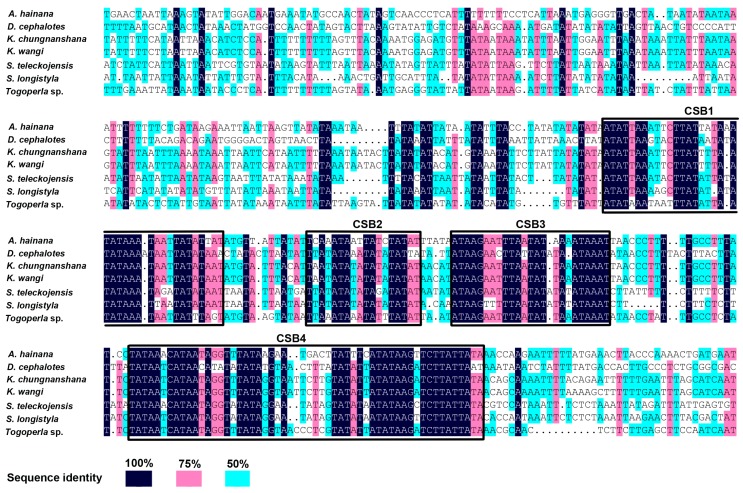
The alignment of conserved structural elements of CRs among stoneflies. Sequence identity among species was indicated by colored boxes. CSB1–4 indicates four conserved sequence blocks in the CRs. Blue color means 100% sequnce identity. Pink color means 75% sequnce identity. Green color means 50% sequnce identity.

Two Stem-loop (SL) structures were predicted in the CR: SL1 (15,445–15,504) and SL2 (15,743–15,790). The proposed stem-loop (SL) structures with a 3′ flanking G(A)_n_T motif were detected after SL1 and SL2, but the 5′ flanking TATA motif was only detected after SL2 (Figure 5B). There was also a 9 bp poly-T stretch (15,464–15,472) near the 5′ end of the CR. In the remainder of the control region, like the *S. longistyla* control region, we also found lots of polynucleotide repeats (≥7 bp) including 2 poly-A, 1 poly-T, and 1 poly-C, which is unusual for a mitogenome control region [31]. When compared with the CRs of the six other stonefly species, four conserved blocks were identified in *S. teleckojensis*: CSB1 (15,726–15,764), CSB2 (15,777–15,794), CSB3 (15,800–15,824), and CSB4 (15,847–15,900) (Figure 6). These CBSs ranged in size from 18 to 54 bp, and their sequence identity among species was generally over 50%. The function of these conserved blocks is unclear. Further study on the mechanistic basis of mtDNA replication is warranted.

### 2.6. Phylogenetic Relationship

**Figure 7:**
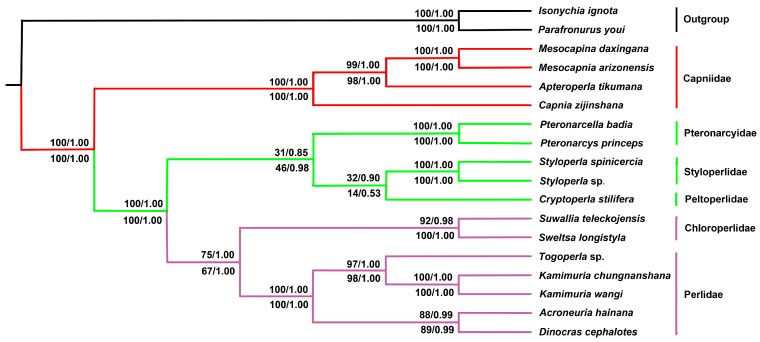
Phylogenetic tree of the 16 sequenced stoneflies. Bayesian inference and Maximum Likelihood Analysis inferred from PCGs and PCGR supported the same topological structure. Values at nodes are ML bootstrap values and Bayesian posterior probabilities using the PCG13 (**up**) and PCGR (**down**) datasets. The tree was rooted with two outgroups (*P. youi* and *I. ignota*).

**Figure A1:**
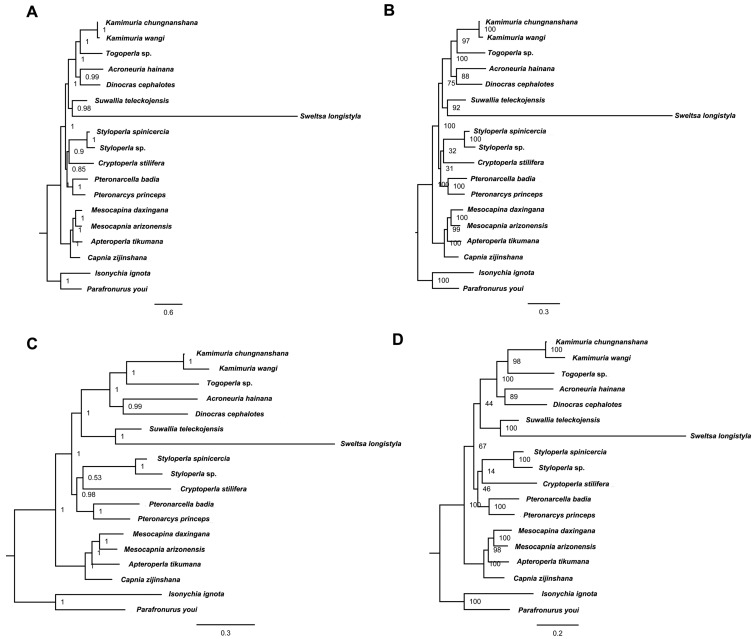
Phylogenetic trees generated from different datasets and methods. (**A**) The tree was obtained by BI analysis of the PCG13 dataset. (**B**) The tree was obtained by ML analysis of the PCG13 dataset. (**C**) The tree was obtained by BI analysis of the PCGR dataset. (**D**) The tree was obtained by ML analysis of the PCGR dataset.

Phylogenetic analyses were performed using nucleotide sequences of 13 PCGs from 16 Plecoptera species, and two Ephemeroptera species were included as the outgroup (Table 1). Bayesian (BI) and maximum likelihood (ML) analyses generated the same tree topologies based on the PCG13 (including 13 PCGs) and PCGR (including 13 PCGs and 2 rRNAs) matrices (Figure 7 and Figure A1). The resultant tree from the BI and ML analyses using PCG13 and PCGR datasets showed strong support for a monophyletic Capniidae, and the other five stonefly families formed a monophyletic group. In this monophyletic group, *S. teleckojensis* was closely related to *S. longistyla* (with bootstrap value of 92/100 and posterior probability of 0.98/1.00 in ML tree and BI tree, respectively), Chloroperlidae and Perlidae were herein corroborated to be a sister group (with bootstrap value of 75/67 and posterior probability of 1.00/1.00 in ML tree and BI tree, respectively). The position of Chloroperlidae and Perlidae is very different from previous studies [20,21,24,25,30]. Instead, our results support the traditional morphology-based classification [26,27]. This phenomenon may result from the limited mitogenomic data, especially for Chloroperlidae species.

Currently, the position of Pteronarcyidae, Styloperlidae, and Peltoperlidae in Pteronarcyoidea has been resolved based on morphology [19,27], but this relationship has not been well supported by molecular data. Two previous studies concluded that Pteronarcyidae was not a sister group of Peltoperlidae [23,25] and Chen et al. (2016) indicated that there was a relationship of Styloperlidae (Pteronarcyidae and Peltoperlidae) [24]. However, the phylogenetic relationship among the three families in Pteronarcyoidea in our study was very different. In this study, Pteronarcyidae was recovered as a sister-group to Styloperlidae and Peltoperlidae with posterior probability of 0.85/0.98 on the tree generated by BI. ML analyses generated the same tree topologies with BI analyses, but the bootstrap value is 31/46 (Figure 7 and Figure A1). These results were generally identical to the recent study made by our previous studies [22] and morphology studies [19,27]. The limited Peltoperlid mitogenomes may result in two clades with low support values and ambiguous relationships among the three families.

The basal position of Capniidae is confirmed and the relationship between Chloroperlidae and Perlidae is supported by BI and ML analyses, but the relationship among Pteronarcyidae, Styloperlidae, and Peltoperlidae remains to be studied in the future. Sampling across more taxonomic levels is very useful and important to gain detailed insights into this problem.

## 3. Materials and Methods 

### 3.1. Specimens and DNA Extraction

A single adult sample of *S. teleckojensis* was used in this study. It was collected from Hanma National Nature Reserve of Inner Mongolia Autonomous Region, China in 2015 with other specimens by Weihai Li. Specimens were preserved in 100% ethanol in the field, and then kept in a −20 °C freezer. The thoracic muscle of the specimen was used for extraction of total genomic DNA using the QIAamp DNA Blood Mini Kit (Qiagen, Germany) and stored at −20 °C until needed. Vouchers consisting of the remaining stoneflies (No. VHem-0021) were deposited in the Entomological Museum of Henan Institute of Science and Technology (HIST), Henan Province, China.

### 3.2. Genome Sequencing, Assembly, and Annotation

The mitogenomes were amplified and sequenced as described in previous studies [22,30,53,54]. The complete mitogenome of *S. teleckojensis* has been deposited in GenBank with accession number MF198253. Sequence reads were assembled into contigs with BioEdit version 7.0.5.3 [55]. tRNA genes were identified by ARWEN with default settings [56]. Two rRNA and all PCG genes were identified by BLAST searches in NCBI (Available online: http://www.ncbi.nlm.nih.gov), and then confirmed by alignment with homologous genes from other published stonefly mitogenomes. The nucleotide composition and codon usage of PCGs were calculated with MEGA 5.0 [57]. Strand asymmetry was measured using the formulas [58]:

AT skew= [A − T]/[A + T]
(1)

GC skew= [G − C]/[G + C]
(2)

The tandem repeats in the putative control region were analyzed with the Tandem Repeats Finder program (Available online: http://tandem.bu.edu/trf/trf.advanced.submit.html) and the stem-loop structures were predicted by Quikfold (Available online: http://unafold.rna.albany.edu/?q=DINAMelt/Quickfold) [59].

### 3.3. Phylogenetic Analysis

Phylogenetic analysis was performed based on 16 complete or nearly complete mitogenomes of stoneflies from GenBank (Table 1). The MAFFT algorithm within the TranslatorX online platform was used to align each PCGs individually [60]. Before back-translating to nucleotides, poorly aligned sites were removed from the protein alignment using GBlocks in the TranslatorX with default settings. We also aligned each rRNA gene individually using the MAFFT v7.0 online server with the G-INS-I strategy [61]. GBlocks v0.91b was used to filter the ambiguous positions in the alignment of rRNAs [62].

The datasets were assembled for our phylogenetic analyses: (1) the “PCG13 matrix” (total of 11,229 bp), including 13 PCGs; (2) the “PCGR matrix” (total of 12,986 bp), including 13 PCGs and 2rRNAs. We selected the best-fit model of nucleotide sequences of each gene by using jModelTest 0.1.1 according to the Akaike Information Criterion (AIC) [63], and the result is shown in Table 5. Bayesian analyses were run with PCG13 and PCGR datasets which were partitioned by gene. The nucleotide matrices were used to reconstruct phylogenetic trees via Bayesian inference (BI) and maximum likelihood (ML) using MrBayes 3.2.6 and RAxML-HPC2 8.1.11, respectively [64,65]. For Bayesian analyses, we conducted two simultaneous runs for 10 million generations. Each set was sampled every 1000 generations with a burn-in rate of 25%. Stationarity was examined by Tracer v.1.5, and was considered to be reached when the ESS (estimated sample size) value was above 200 [66]. For ML analyses of nucleotide sequences, bootstrapping analyses with 1000 replicates were performed with the fast ML method implemented in RAxML using the GTRGAMMA model.

## Figures and Tables

**Table 1:** General informatics of the taxa used in this study.

Taxonomic Order	Family	Species	Number (bp)	Accession Number
Plecoptera	Perlidae	*Acroneuria hainana*	15,804	NC_026104
		*Togoperla* sp.	15,723	KM409708
		*Kamimuria wangi*	16,179	NC_024033
		*Kamimuria chungnanshana*	15,943	NC_028076
		*Dinocras cephalotes*	15,666	NC_022843
	Pteronarcyidae	*Pteronarcys princeps*	16,004	NC_006133
		*Pteronarcella badia*	15,585	NC_029248
	Capniidae	*Apteroperla tikumana*	15,564	NC_027698
		*Capnia zijinshana*	16,310	KX094942
		*Mesocapnia arizonensis*	14,921	KP642637 *
		*Mesocapnia daxingana*	15,524	KY568983 *
	Peltoperlidae	*Cryptoperla stilifera*	15,633	KC952026 *
		*Soliperla* sp.	15,877	MF716958
	Styloperlidae	*Styloperla* sp.	15,416	KR088971 *
		*Styloperla spinicercia*	16,129	KX845569
	Chloroperlidae	*Sweltsa longistyla*	16,151	KM216826
		*Suwallia teleckojensis*	16,146	MF198253
	Taeniopterygidae	*Taeniopteryx ugola*	15,353	MG589786
		*Doddsia occidentalis*	16,020	MG589787
	Gripopterygidae	*Zelandoperla fenestrata*	16,385	KY522907
	Perlodidae	*Perlodes* sp.	16,039	MF197377
		*Isoperla bilineata*	15,048	MF716959
Ephemeroptera	Heptageniidae	*Parafronurus youi*	15,481	EU349015
	Isonychiidae	*Isonychia ignota*	15,105	HM143892

* Incomplete genome sequence.

**Table 2:** Organization of the *S. teleckojensis* mitochondrial genome.

Gene	Direction	Location	Size	Anticodon	Anticodon Positions	Start/Stop Codons	Intergenic Nucleotides
*tRNA^Ile^*	Forward	1–67	67	GAT	30–32		
*tRNA^Gln^*	Reverse	65–133	69	TTG	101–103		−3
*tRNA^Met^*	Forward	135–203	69	CAT	165–167		1
*ND2*	Forward	204–1238	1035			ATG/TAA	0
*tRNA^Trp^*	Forward	1237–1305	69	TCA	1267–1269		−2
*tRNA^Cys^*	Reverse	1298–1364	67	GCA	1331–1333		−8
*tRNA^Tyr^*	Reverse	1367–1433	67	GTA	1398–1400		2
*COI*	Forward	1426–2982	1557			ATT/TAA	−8
*tRNA^Leu(UUR)^*	Forward	3002–3067	66	TAA	3031–3033		19
*COII*	Forward	3081–3768	688			ATG/T-	13
*tRNA^Lys^*	Forward	3769–3839	71	CTT	3799–3801		0
*tRNA^Asp^*	Forward	3839–3907	69	GTC	3869–3871		−1
*ATP8*	Forward	3908–4066	159			ATG/TAA	0
*ATP6*	Forward	4060–4737	678			ATG/TAA	−7
*COIII*	Forward	4737–5525	789			ATG/TAA	−1
*tRNA^Gly^*	Forward	5528–5593	66	TCC	5558–5560		2
*ND3*	Forward	5594–5947	354			ATC/TAG	0
*tRNA^Ala^*	Forward	5946–6011	66	TGC	5975–5977		−2
*tRNA^Arg^*	Forward	6013–6077	65	TCG	6042–6044		1
*tRNA^Asn^*	Forward	6082–6147	66	GTT	6112–6114		4
*tRNA^Ser(AGN)^*	Forward	6148–6214	67	GCT	6173–6175		0
*tRNA^Glu^*	Forward	6215–6281	67	TTC	6246–6248		0
*tRNA^Phe^*	Reverse	6287–6351	65	GAA	6320–6322		5
*ND5*	Reverse	6352–8086	1735			GTG/T-	0
*tRNA^His^*	Reverse	8087–8154	68	GTG	8121–8123		0
*ND4*	Reverse	8159–9499	1341			ATG/TAA	4
*ND4L*	Reverse	9493–9789	297			ATG/TAA	−7
*tRNA^Thr^*	Forward	9792–9859	68	TGT	9822–9824		2
*tRNA^Pro^*	Reverse	9861–9928	68	TGG	9896–9898		1
*ND6*	Forward	9930–10,454	525			ATC/TAA	1
*CytB*	Forward	10,454–11,590	1137			ATG/TAA	−1
*tRNA^Ser(UCN)^*	Forward	11,590–11,659	70	TGA	11,621–11,623		−1
*ND1*	Reverse	11,680–12,630	951			TTG/TAG	20
*tRNA^Leu(CUN)^*	Reverse	12,632–12,697	66	TAG	12,666–12,668		1
*lrRNA*	Reverse	12,698–14,026	1329				0
*tRNA^Val^*	Reverse	14,027–14,097	71	TAC	14,062–14,064		0
*srRNA*	Reverse	14,098–14,897	800				0
*CR*		14,898–16,146	1249				0

**Table 3:** The nucleotide composition of the *S. teleckojensis* mitogenome.

Feature	Proportion of Nucleotides							No. of Nucleotides
	%T	%C	%A	%G	%A + T	AT Skew	GC Skew	
Whole genome	30.2	21.7	36.5	11.6	66.7	0.09	−0.303	16,146
PCGs	37.6	18.9	26.5	17.0	64.1	−0.17	−0.052	11,244
First codon position	40.6	20.7	21.9	16.9	62.4	−0.30	−0.102	3743
Second codon position	37.5	18.1	28.5	15.9	66.0	−0.14	−0.063	3743
Third codon position	34.8	17.8	29.1	18.2	64.0	−0.09	0.011	3743
PCG-J	32.9	24.5	28.9	13.7	61.8	−0.07	−0.281	6921
First codon position	36.4	23.1	24.5	16.0	60.9	−0.20	−0.182	2307
Second codon position	32.0	27.2	30.4	10.3	62.4	−0.03	−0.450	2307
Third codon position	30.4	23.0	31.8	14.8	62.1	0.02	−0.217	2307
PCG-N	45.1	9.9	22.7	22.2	67.8	−0.33	0.383	4323
First codon position	41.9	9.5	25.0	23.6	66.9	−0.25	0.427	1441
Second codon position	47.1	16.9	17.8	18.3	64.9	−0.45	0.040	1441
Third codon position	46.4	3.4	25.4	24.8	71.8	−0.29	0.759	1441
tRNA genes	34.6	13.9	34.6	16.9	69.2	0.00	0.100	1487
tRNA-J	33.8	15.1	36.2	14.9	70.0	0.03	−0.007	946
tRNA-N	36.0	11.6	31.8	20.5	67.8	−0.06	0.276	541
rRNA genes	39.9	9.4	31.6	19.1	71.5	−0.12	0.338	2129
lrRNA	40.3	8.3	32.9	18.5	73.2	−0.10	0.382	1329
srRNA	39.1	11.4	29.5	20.0	68.6	−0.14	0.275	800
CR	35.5	13.3	43.6	7.6	79.1	0.10	−0.272	1249

**Table 4:** Codon usage of *S. teleckojensis* mitochondrial genome protein-coding genes.

Amino Acid	Codon	N	RSCU	N+	RSCU	N−	RSCU
Phe (F)	UUU	208	1.36	85	0.98	123	1.86
	UUC	98	0.64	89	1.02	9	0.14
Leu (L)	UUA	280	2.6	131	2.13	149	3.23
	UUG	108	1	12	0.2	96	2.08
	CUU	67	0.62	53	0.86	14	0.3
	CUC	67	0.62	66	1.07	1	0.02
	CUA	112	1.04	97	1.58	15	0.32
	CUG	12	0.11	10	0.16	2	0.04
Ile (I)	AUU	213	1.46	133	1.29	80	1.86
	AUC	79	0.54	73	0.71	6	0.14
Met (M)	AUA	141	1.45	100	1.72	41	1.04
	AUG	54	0.55	16	0.28	38	0.96
Val (V)	GUU	82	1.38	29	0.88	53	2.02
	GUC	44	0.74	37	1.12	7	0.27
	GUA	70	1.18	55	1.67	15	0.57
	GUG	41	0.69	11	0.33	30	1.14
Ser (S)	UCU	94	2.32	39	1.72	55	3.08
	UCC	30	0.74	29	1.28	1	0.06
	UCA	63	1.56	48	2.12	15	0.84
	UCG	15	0.37	7	0.31	8	0.45
Pro (P)	CCU	59	1.55	34	1.21	25	2.5
	CCC	48	1.26	45	1.61	3	0.3
	CCA	36	0.95	29	1.04	7	0.7
	CCG	9	0.24	4	0.14	5	0.5
Thr (T)	ACU	66	1.25	43	1.07	23	1.8
	ACC	59	1.11	58	1.44	1	0.08
	ACA	73	1.38	56	1.39	17	1.33
	ACG	14	0.26	4	0.1	10	0.78
Ala (A)	GCU	84	1.51	48	1.29	36	1.97
	GCC	76	1.37	72	1.93	4	0.22
	GCA	37	0.67	27	0.72	10	0.55
	GCG	25	0.45	2	0.05	23	1.26
Tyr (Y)	UAU	106	1.39	32	0.84	74	1.95
	UAC	46	0.61	44	1.16	2	0.05
Stop (*)	UAA	9	1.64	7	1.75	2	1.33
	UAG	2	0.36	1	0.25	1	0.67
His (H)	CAU	46	1.12	30	0.91	16	2
	CAC	36	0.88	36	1.09	0	0
Gln (Q)	CAA	70	1.71	57	1.97	13	1.08
	CAG	12	0.29	1	0.03	11	0.92
Asn (N)	AAU	110	1.44	63	1.19	47	2
	AAC	43	0.56	43	0.81	0	0
Lys (K)	AAA	35	1.03	29	1.66	6	0.36
	AAG	33	0.97	6	0.34	27	1.64
Asp (D)	GAU	51	1.4	31	1.19	20	1.9
	GAC	22	0.6	21	0.81	1	0.1
Glu (E)	GAA	55	1.34	43	1.87	12	0.67
	GAG	27	0.66	3	0.13	24	1.33
Cys (C)	UGU	38	1.69	7	1	31	2
	UGC	7	0.31	7	1	0	0
Trp (W)	UGA	82	1.56	63	1.85	19	1.03
	UGG	23	0.44	5	0.15	18	0.97
Arg (R)	CGU	13	0.81	4	0.41	9	1.44
	CGC	2	0.13	2	0.21	0	0
	CGA	33	2.06	29	2.97	4	0.64
	CGG	16	1	4	0.41	12	1.92
Ser (S)	AGU	49	1.21	22	0.97	27	1.51
	AGC	18	0.44	12	0.53	6	0.34
	AGA	52	1.28	24	1.06	28	1.57
	AGG	3	0.07	0	0	3	0.17
Gly (G)	GGU	56	0.91	21	0.6	35	1.32
	GGC	22	0.36	14	0.4	8	0.3
	GGA	92	1.5	79	2.27	13	0.49
	GGG	75	1.22	25	0.72	50	1.89

N: Total number in all PCGs, N+: total number in J-strand, N−: total number in N-strand, RSCU: relative synonymous codon usage.

**Table 5:** Best evolutionary models selected by jModelTest.

Gene	Best Model
*ATP6*	GTR + I + G
*ATP8*	GTR + G
*COI*	GTR + G
*COII*	GTR + G
*COIII*	GTR + I + G
*CytB*	GTR + G
*ND1*	GTR + I + G
*ND2*	GTR + I + G
*ND3*	GTR + I + G
*ND4*	GTR + I + G
*ND4L*	GTR + I + G
*ND5*	GTR + I + G
*ND6*	GTR + I + G
*srRNA*	GTR + I + G
*lrRNA*	GTR + G

## Abbreviations

mtDNA	Mitochondrial DNA
PCG	Protein-Coding Gene
tRNA	Transfer RNA
rRNA	Ribosomal RNA
CR	Control Region
SL	Stem-Loop
CSB	Conserved Sequence Block
ML	Maximum Likelihood
BI	Bayesian Inferences
